# The role of chaperone-mediated autophagy in drug resistance

**DOI:** 10.1590/1678-4685-GMB-2023-0317

**Published:** 2024-05-31

**Authors:** Ana Beatriz da Silva Teixeira, Maria Carolina Clares Ramalho, Izadora de Souza, Izabela Amélia Marques de Andrade, Isabeli Yumi Araújo Osawa, Camila Banca Guedes, Beatriz Silva de Oliveira, Cláudio Henrique Dahne de Souza, Tainá Lins da Silva, Natália Cestari Moreno, Marcela Teatin Latancia, Clarissa Ribeiro Reily Rocha

**Affiliations:** 1 Universidade Federal de São Paulo (UNIFESP), Departamento de Oncologia Clínica e Experimental, São Paulo, SP, Brazil.; 2 National Institutes of Health, National Institute of Child Health and Human Development, Laboratory of Genomic Integrity, Bethesda, MD, USA.

**Keywords:** Chaperone-mediated autophagy, cancer, resistance

## Abstract

In the search for alternatives to overcome the challenge imposed by drug resistance development in cancer treatment, the modulation of autophagy has emerged as a promising alternative that has achieved good results in clinical trials. Nevertheless, most of these studies have overlooked a novel and selective type of autophagy: chaperone-mediated autophagy (CMA). Following its discovery, research into CMA’s contribution to tumor progression has accelerated rapidly. Therefore, we now understand that stress conditions are the primary signal responsible for modulating CMA in cancer cells. In turn, the degradation of proteins by CMA can offer important advantages for tumorigenesis, since tumor suppressor proteins are CMA targets. Such mutual interaction between the tumor microenvironment and CMA also plays a crucial part in establishing therapy resistance, making this discussion the focus of the present review. Thus, we highlight how suppression of LAMP2A can enhance the sensitivity of cancer cells to several drugs, just as downregulation of CMA activity can lead to resistance in certain cases. Given this panorama, it is important to identify selective modulators of CMA to enhance the therapeutic response.

## Introduction

Cancer encompasses a diverse range of approximately 277 distinct disease types (Demir Cetinkaya and Biray Avci, 2022), and it is one of the most extensively studied conditions in any area of science. The clonal evolutionary concept of cancer progression was introduced in 1976. This model essentially posits that genetically unstable cells display several gene mutations, and selective pressures - arising from both endogenous or exogenous factors, such as chemotherapeutic treatment - foster the growth and survival of subpopulations possessing a biological fitness advantage (Nowell, 1976). This phenomenon contributes to the molecular complexity of cancer and the pronounced heterogeneity found within each type of cancer. Certainly, this divergency lies at the core of the challenge in achieving high effectiveness in healing and therapeutic processes for patients, primarily due to acquired resistance achieved by natural selection during cancer development. Therefore, cancer ranks as the second leading global cause of death among children and adults worldwide (Naghavi et al., 2017), and its prevalence is on the rise.

In fact, cancer poses a significant health challenge for humanity as a whole, prompting extensive studies majorly focusing on therapeutic drug development. However, one of the critical hurdles encountered during cancer therapy is the emergence of resistance mechanisms. Some examples of resistance mechanisms that cancer cells can employ are alterations of efflux or influx pumping of the drug to the cell, DNA repair pathways activation, immune evasion, and metabolic adaptation (Holohan et al., 2013). Thus, given that cancer cells exhibit abnormal nutrient requirements in contrast to normal cells, pathways that control cell metabolism and growth are an interesting target to be studied as potential therapeutic alternatives and ways to overcome resistance. In this context, the autophagy process, which involves the cellular recycling of components, has gained significant attention over the past years. Modulating autophagic pathways present a promising avenue for targeted cancer therapies, as it can influence the survival and growth of cancer cells by controlling their nutrient use and stress responses.

Given the substantial data regarding the role of autophagy in supporting tumor growth and promote resistance (Levy et al., 2017; Mele et al., 2020; Noman et al., 2020), researchers are actively investigating how to modulate it to enhance and overcome antitumor treatments, with promising results emerging from clinical trials, but also United States Food and Drug Administration (FDA) approved agents (reviewed in Mohsen et al., 2022). Until now, the only FDA-approved autophagy inhibitors are Chloroquine and Hydroxychloroquine, both being used to treat Malaria (Ganguli et al., 2014; Manic et al., 2014; De Sanctis et al., 2023). A more novel pathway of the NEDD-8 inhibitory agent, Pevonedistat, is undergoing clinical trials. Pevonedistat (MLN4924) has been shown to increase cell apoptosis and autophagy via neddylation (Soucy et al., 2010). Therefore, unlike Chloroquine/Hydroxychloroquine, Pevonedistat acts as a pro-apoptotic and autophagy inducer rather than an inhibitor.

Considering these aforementioned developments, there is a substantial expanse of this yet unexplored field towards the domain of autophagy modulation, targeting an enhanced response to cancer treatment. Nevertheless, the ongoing trials are primarily oriented towards the modulation of only one form of autophagy, known as macroautophagy (MA) (Deretic, 2008; Hu et al., 2008) overlooking a very important form of autophagy named Chaperone-Mediated Autophagy (CMA). Such limited approach accentuates the vast prospects for advancing cancer treatment through a comprehensive understanding of these complex pathways, that we aim to better explore in this review.

### Discovery of selective lysosomal degradation: CMA

In 1963, Christian de Duve used the term “autophagy”, derived from the Greek for “self-eating”, to describe the presence of single or double-membrane vesicles containing pieces of cytoplasm and organelles in various states of degradation (De Duve and Wattiaux, 1966). Currently, it is known that autophagy is a mechanism by which cytoplasmic material is delivered to the lysosome for degradation and recycling, being a crucial physiological process for sustaining eukaryotic cells homeostasis (Ohsumi, 2014; Parzych and Klionsky, 2014). Different from the ubiquitin-proteasome system (UPS) (Nandi et al., 2006), autophagy is not limited to protein degradation, but also plays a central role in cell metabolism, in addition to the degradation of other types of biomolecules such as carbohydrates and lipids, organelles and even some pathogens (Parzych and Klionsky, 2014). Thus, autophagy is related to several biological events, which occur in response to stressful situations but also under physiological conditions to support normal cellular functions. Some of these include catabolizing the degradation and recycling of intracellular components by the elimination of defective proteins and organelles assuring nutrient and energy balance, cellular stress response, prevention of abnormal protein aggregate accumulation, removal of intracellular pathogens, and DNA repair and cell death (Galluzzi et al., 2017).

The fundamental significance of autophagy for health and longevity has been confirmed by numerous studies suggesting that various autophagic processes are compromised with aging (Kaushik et al., 2021). Moreover, autophagy has been linked to several human health conditions, including cancer (Ogier-Denis and Codogno, 2003; White, 2015). As autophagy can promote cell survival during stress conditions, in the context of cancer treatment, this process can have a dual role, either pro-survival or pro-death, depending on the different stages of tumorigenesis. On the one hand, if by breaking down cellular components and recycling them, cancer cells can sustain their energy needs and reduce the toxic effects of treatment, enabling them to survive and recover (Amaravadi, 2008; Laddha et al., 2014; Strohecker and White, 2014; Guo et al., 2016; Yamamoto et al., 2020), on the other hand, excessive or prolonged autophagy can lead to autophagic programmed cell death, contributing to treatment-induced cell death (Liang et al., 1999; Qu et al., 2003; Mathew et al., 2009).

As mentioned previously, the understanding of the mechanism and pathophysiology of autophagy has expanded over the last few decades. Initially, the term ‘autophagy’ was used to refer only to MA, but we now know that it refers to three distinct types, named: MA, CMA and Microautophagy (MI) (Duan and Tong, 2021). Although these types share a commonality in degrading damaged cellular components through lysosomes, their approaches to transporting substrates to the lysosome differ greatly (Yang and Klionsky, 2010).

In mammals, MA is the most comprehensively researched process and it starts with the formation of a double-membrane vesicle known as an autophagosome that absorbs molecules or organelles found in the cytoplasm. It then merges with the lysosome giving rise to the autolysosome, resulting in the cargo degradation (Feng et al., 2014). Microautophagy is the pathway that has received the least attention. It works by invaginating a portion of the cytoplasm through deformation of the lysosomal membrane (Mijaljica et al., 2011; Parzych and Klionsky, 2014). These two pathways function by simultaneously sequestering different cytosolic components, thus lacking the capacity to degrade biomolecules selectively and individually.

Unlike the other types of autophagy pathways, CMA is a selective form specialized in protein degradation, which is based on its individual translocation through the lysosomal membrane after recognizing a specific sequence motif (KFERQ-like) (Orenstein and Cuervo, 2010; Kaushik and Cuervo, 2018; Auzmendi-Iriarte and Matheu, 2021). Importantly, approximately 40% of cytosolic proteins contain the amino acid sequence corresponding to this motif (Tekirdag and Cuervo, 2018). Structurally, KFERQ refers to a pentapeptide sequence containing a lysine residue; one of the four hydrophobic amino acids (phenylalanine, valine, leucine or isoleucine); glutamic acid or aspartic acid; arginine at the beginning or end of the sequence; glutamine (Fred Dice, 1990). Post-translational modifications, like phosphorylation or acetylation, can provide the necessary charge and complete the motif, even if only four of the five amino acids are present in the protein by constitution (Bandyopadhyay et al., 2008).

**Figure 1 -:**
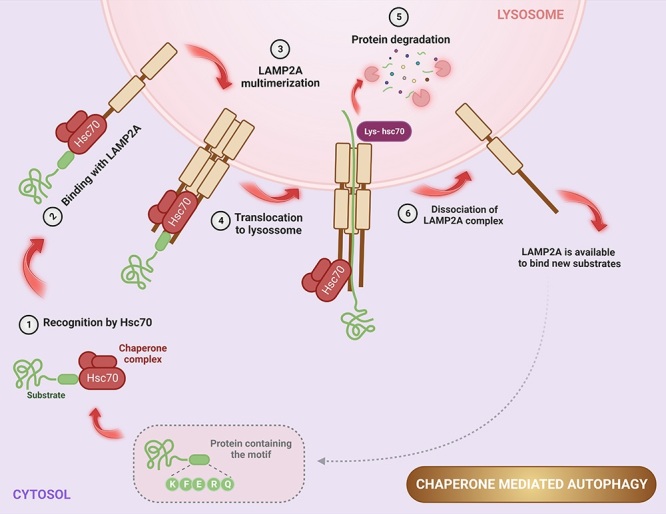
CMA mechanism. The CMA pathway is initiated by recognition of the target protein in the cytosol by binding of the Hsc70 chaperone to the KFERQ motif present in the substrate (1). Subsequently, the substrate is directed to the surface of the lysosome membrane and encounters LAMP2A (2), which forms a multimeric complex (3), allowing protein translocation into the lysosomal lumen (4). Upon complete protein degradation (5), LAMP2A returns to its monomeric state (6) to initiate new processes and translocations. Created with BioRender.

Of notice, the involvement of co-chaperones is a crucial aspect for the CMA mechanism (Figure 1) to function efficiently, as they play a key role in regulating other chaperones that are necessary for the lysosomal degradation process (Agarraberes and Dice, 2001). In the cytosol, substrate recognition is carried out by the 70 kDa heat shock cognate protein (Hsc70 chaperone), also known as HSPA8 (heat shock protein family A [Hsp70] member 8). Hsc70 binds to the KFERQ-like motif present in the target protein (Chiang et al., 1989), and the complex is subsequently targeted to the surface of the lysosomal membrane. At this stage, the lysosome-associated membrane protein type 2A (LAMP2A), a receptor for CMA substrates (Cuervo and Dice, 1996), binds to the substrate and multimerizes to enable its translocation into the lysosomal lumen (Bandyopadhyay et al., 2008). Meanwhile, the chaperone Hsc90 maintains the stability of LAMP2A and prevents the target proteins from refolding and hindering transport. After substrate-receptor binding, the chaperone present in the lysosomal lumen (lys-hsc70) helps the cargo translocation for degradation. After complete substrate degradation in the lysosomal matrix, LAMP2A can return to its initial state and bind to new proteins, allowing the translocation and degradation cycle to continue (Agarraberes and Dice, 2001; Orenstein and Cuervo, 2010).

Thus, *de novo* synthesis of LAMP2A is not mandatory for the initiation of a new degradation cycle. The lysosomal abundance of this protein can be modulated by changes in its stability, organization and dynamics in the lysosomal membrane, properties orchestrated by proteins associated with the lysosome, such as the GFAP/EF1α pair and the mTORC2/AKT1/PHLPP1 axis (Arias and Cuervo, 2020). Importantly, the LAMP2A protein is essential for the proper functioning of the CMA, as several studies have identified it as the key protein in the pathway. As a consequence, the levels of LAMP2A present in the lysosomal membrane regulate the rate of CMA performance (Park et al., 2015). Hence, modulation of LAMP2A is one of the methods used to study CMA. 

### All eyes on the CMA: Experimental approaches to this selective autophagy

Despite the well-defined specificities of CMA in relation to the other types of autophagy, one must consider that in most cells, including tumor cells, there is a crosstalk between the three autophagic pathways and there may be compensation between them in the event of deficiency or malfunction (Massey et al., 2006; Schneider et al., 2015). Because of this extensive connection between the different autophagic pathways, specific methods for assessing CMA activity are needed in order to understand its role, whether in a pathological or physiological condition or in response to possible therapeutic interventions (Arias and Cuervo, 2020). Initially, the search for methods to assess and measure CMA was more complex than for MA because the structural characteristics at the molecular level were not fully understood. But as demonstrated, there is now a foundation for a wide range of methods to study CMA, providing new tools to help researchers in the search for CMA modulation as a therapeutic target (Hubert et al., 2022). The evaluation methods for CMA are categorized as shown in Table 1. It is noteworthy that some techniques have intrinsic limitations and may require additional complementary methods.

**Table 1 -:** Experiments for CMA evaluation.

Method	Purpose	Limitations
LAMP2A immunoblotting and imaging	Analyze changes in key CMA components and indirectly measure CMA functionality.	The presence of LAMP2A doesn’t predict functional CMA, only when the lysosomal subpopulations contain LAMP2A and lys-hsc70.
Pulse and chase experiments*	Evaluation of intracellular protein degradation assessment	Endosomal microautophagy may remain unaffected by 3MA and contribute to lysosomal proteolysis. Increased protein degradation via CMA due to MA inactivation.
Photoconvertible CMA reporters photoswitchable and photoactivable	Observe the lysosomal association of artificial fluorescent CMA reporters	Alterations in degradation within the lysosomal compartment remain undetectable.
*In vitro* reconstitution of CMA with isolated lysosomes	Measure functional CMA	Endosomal microautophagy may remain unaffected by 3MA and contribute to lysosomal proteolysis. Alterations in degradation within the lysosomal compartment remain undetectable. Increased protein degradation via CMA due to MA inactivation. Modifying CMA in cells might influence other autophagic pathways.

*Radiolabeled amino acid and inhibitors of either lysosomal proteases or other autophagic pathways.

The most frequent methods used to study changes in CMA activity are immunoblotting and imaging, in which antibodies that can specifically recognize LAMP2A distinguishing from its variants LAMP2B and LAMP2C are used. These experiments, known as *steady-state assays*, can evaluate the overall activity of CMA. This category includes fluorescence assays, the use of immunogold experiments in tissues to quantify the presence of CMA activity within lysosomes, and the evaluation of lysosomal levels of CMA components (such as hsc70 and LAMP2A). There are many assays available to measure CMA activity. One of the strategies is the evaluation of lysosomal levels of LAMP2A (colocalized with hsc70 and associated with Lys-HSC70), since increased rates of CMA often correlates with high levels of LAMP2A (Cuervo and Dice, 1996; Agarraberes and Dice, 2001). However, functional assays, such as intracellular protein degradation, photoconvertible CMA reporters, and isolated lysosome *in vitro*/*in vivo* methods provide a more accurate examination of CMA activity over time (Patel and Cuervo, 2015).

Besides that, pulse and chase experiments are very powerful tools to measure CMA, as it uses a radiolabeled amino acid together with inhibitors that target lysosomal proteases or alternative autophagic pathways (like MA) (Kaushik and Cuervo, 2018). Thus, if protein degradation is more sensitive to lysosome inhibitors such as leupeptin and ammonium chloride (NH4Cl) and insensitive to MA inhibitors such 3-MA, it is considered to be a CMA-dependent process (Fuertes et al., 2003). 

## The dual role of CMA in cancer

Physiologically, CMA is responsible for the degradation of misfolded and damaged proteins preventing cellular proteotoxicity (Jackson and Hewitt, 2016; Kaushik and Cuervo, 2018). This autophagic pathway, like the others, is activated by stressors such as hypoxia, oxidative stress, tissue remodeling, nutrient deprivation and in response to genotoxic insults (Kiffin et al., 2004; Park et al., 2015). Therefore, impairment of the degradation of specific substrates by CMA may alter several cellular processes resulting, for instance, in cell sensitivity, genomic instability and defects in DNA maintenance and repair (Gomes et al., 2017 b ).

Interestingly, several studies have shown that CMA can act as a tumor suppressor, preserving genomic stability and regulating the levels of proto-oncogenic (Gomes et al., 2017 a ; Arias and Cuervo, 2020), thus preventing malignant transformation. On the other hand, in tumor cells, CMA may act as an important precursor of tumorigenesis: it is known that certain features of the tumor microenvironment can stimulate the positive regulation of CMA, resulting in the modulation of proteins important for cancer development (Han et al., 2017; Arias and Cuervo, 2020). Therefore, although CMA plays a crucial role in cells in healthy conditions, in the oncogenic context, which is the focus of this review, modulation of this pathway is observed in tumor development, survival and progression (Kon et al., 2011).

## What regulates CMA

Tumor cells often adapt to cope with environments featuring stress signaling. In fact, tumor microenvironment imposes a variety of challenges for cells, including hypoxia, a scarcity of growth factors and particular nutrients, and weakened substrate adhesion. Consequently, over time, only the best adjusted cells will survive, a process usually referred as a “potentiated state” (Arias and Cuervo, 2020). Importantly, stress conditions are the primary signal responsible for the activation of CMA (Kaushik and Cuervo, 2018).

In this sense, one of the most well-documented stress situations related to CMA activation is nutrient restriction. Typically, MA is triggered in the initial hours following serum removal, while CMA activity progressively rises, peaking around 10 hours post serum withdrawal in cultured cells or 3 days in animals (Li et al., 2011). One of the pioneering studies addressing this topic was carried out by Cuervo’s group, reporting the activation of a selective metabolic pathway for lysosomal proteolysis in rat livers when subjected to extended food restriction (Cuervo *et al.,* 1995). Such findings are important once cells might benefit by transitioning to a more selective degradation, allowing essential proteins to remain in the cytosol while targeting less critical ones for breakdown (Orenstein and Cuervo, 2010).

Another documented stressor that can activate CMA is hypoxia, a condition characterized by inadequate oxygen availability in tissues, leading to a range of adaptive responses in cells, among them autophagy. The triggering of CMA during hypoxia may assist cells in selectively regulating the protein pool, removing potentially harmful or unnecessary proteins while preserving those essential for adaptation and survival under low oxygen conditions (Daskalaki et al., 2018). Moreover, other stress signals, such as DNA damage, are crucial for CMA activation and it has been shown that a failure in its activation can lead to the accumulation of damage (Park et al., 2015).

Finally, the redox status of cells governs CMA activity, which is believed to be an important mechanism to remove oxidized proteins. Additionally, the existing evidence suggests that the application of antioxidants can, to some extent or entirely, reverse or modulate autophagy, emphasizing the role of CMA in the elimination of oxidized proteins (Levonen et al., 2014). NRF2 (nuclear factor erythroid 2-related factor 2) is a protein that plays a pivotal role in regulating the cellular response to stress, especially oxidative stress, a primary form of stress to which tumor cells are subjected. Under physiological conditions, NRF2 is bound to a protein named KEAP-1, which tags it for degradation. In the presence of oxidative stress, NRF2 dissociates from Kelch‐like ECH‐associated protein 1 (KEAP1), translocates into the cell nucleus, and binds to antioxidant response elements (AREs) (De Souza et al., 2022; Almeida Lima et al., 2023). It has been shown that NRF2 binds to the AREs in the LAMP2 gene, regulating its cellular levels. As a result, both overexpression of NRF2 or its pharmacological activation led to increased levels of LAMP2A and, subsequently, higher CMA activity. In the same study, the authors demonstrated that in mice knocked out for NRF2, CMA was impaired in lysosomes (Pajares et al., 2018).

Recently, another study highlighted the role of NRF2 in the activation of CMA. In essence, the authors demonstrate the formation of an NRF2-CMA axis in the form of a positive feedback loop to enhance the antioxidant response and protect cells, achieved through a CMA-depended KEAP1 degradation. The main novelty presented in the study is that CMA regulates NRF2, which combined with previous findings, supports the conclusion that this regulation is reciprocal (Zhu et al., 2022).

It is important to note that CMA activity can also be regulated by several signaling pathways that may be altered in the context of cancer (Hubert et al., 2022). The first signaling mechanism identified in CMA activation was the NFAT (calcium-regulated phosphatase) pathway, which provided unique insights into CMA activation in response to oxidative stress. During T cell activation, the production of reactive oxygen species (ROS) stimulates the transcription factor NFAT1 to bind to LAMP2 proximal promoter region causing the upregulation of LAMP2A (Valdor et al., 2014). Another example is the Endoplasmic reticulum (ER) stress-induced activation of the p38 MAPK signaling pathway leading to a dual phosphorylation of LAMP2A which activates CMA, termed the ERICA pathway for “ER-stress-induced CMA” (Li et al., 2017). In addition, Anguiano and coworkers showed the transcriptional inhibition of LAMP2A through the signaling of retinoic acid receptor alpha (RARα) (Anguiano *et al*., 2013). Also, the mTORC2/AKT1/PHLPP1 axis coordinates the dynamic assembly and disassembly of LAMP2A into multimers, through the phosphatase PHLPP1 inhibition of mTORC2 function, thereby blocking the activation of AKT and promoting the formation of LAMP2A multimers (Arias et al., 2015). Finally, a lysosome-associated form of the GFAP (glial fibrillary acidic protein) and EF1α (elongation factor 1α) also modulated CMA activity in response to oxidative stress (Assaye and Gizaw, 2022).

In light of this, it becomes clear that the regulation of CMA serves as a pivotal response mechanism to various stress conditions, particularly those prevalent in tumoral environments, rather than genetic alterations in the CMA machinery itself. In fact, mutations in the LAMP2 gene, which encodes the LAMP2A protein, have only been associated with Danon disease, a severe condition that is characterized by skeletal and cardiac myopathy, as well as cognitive impairment, and no increase in cancer susceptibility (Morell et al., 2016). In contrast, genetic alterations in pathways that control CMA, mentioned above, are frequently found in cancer. These include somatic NRF2 and KEAP1 mutations, hypermethylation of KEAP1 and amplification of NRF2, which culminates in a constitutive NRF2 activation; gain-of-function mutations of the canonical transient receptor potential channel (TRPC6) that leads to enhanced NFAT signaling; mutations or overexpression of genes that regulate p38 MAPK activity; chromosomal translocations involving the RARα locus; and mutations on a subunit required for mTORC2 activity (mLST8) that leads to oncogenic mTORC2-AKT activation (Parrado et al., 2000; Pan et al., 2013; Pouremamali et al., 2022; Chen et al., 2023). However, even though mutations in these pathways have the potential to consequently lead to alterations in CMA activity, there is still no clear evidence linking these mutations to their impact on CMA in cancer cells. 

**Figure 2 -:**
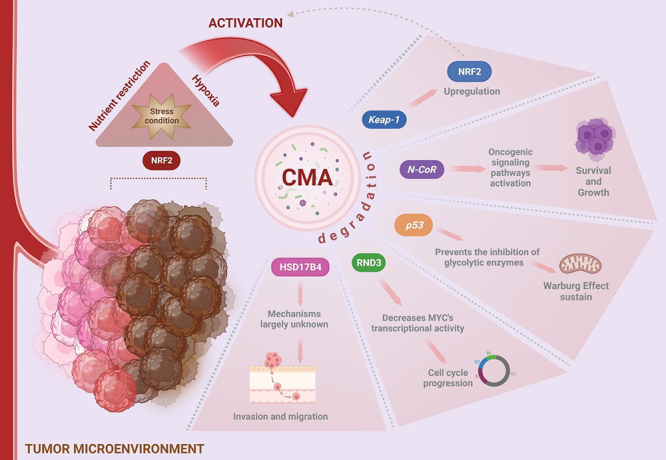
CMA is influenced by and regulates the tumor microenvironment. The stress condition of the tumor microenvironment is the primary signal for the activation of CMA. Specifically, nutrient restriction, hypoxia and NRF2 pathway are well-documented CMA activators. On the other hand, CMA degrades important proteins such as KEAP-1, N-Cor, p53, RND3 and HSD17B4, which supports the tumor microenvironment, resulting in the progression of cancer by increasing NRF2, promoting cell survival and growth, sustaining the Warburg effect, regulating the cell cycle, and promoting cell invasion and migration. Created with BioRender.

In general, alterations in CMA principal components observed in cancer are associated with changes in Hsc70 and LAMP2A protein and mRNA levels, not mutations in their genes (Rios et al., 2021). And when those levels are found elevated in malignant cells indicating that CMA is activated, this type of autophagy can degrade key proteins required for tumor growth and development. Thus, in addition to being influenced by the tumor microenvironment, CMA is also capable of regulating it (Figure 2).

## What CMA regulates

The selectivity of CMA in degrading specific proteins involved in several cellular processes confers regulatory function to this autophagic pathway (Tekirdag and Cuervo, 2018). Notably, in the context of cancer, multiple CMA substrate proteins were found to be deregulated in a wide range of cancer cell lines (Figure 2). It has been reported that upregulation of CMA favors the survival and proliferation of cancer cells, promoting tumor growth (Kon et al., 2011; Saha, 2012) and that enhanced CMA activity is a common feature among different cancer cell lines and human tumors independently of the MA status. In addition, several studies have shown that CMA has a pro-oncogenic function by helping the cancer cells to cope with stress conditions often found in the tumor microenvironment, and higher energetic demand supported by aerobic glycolysis to sustain increased proliferative capacity (Kon *et al*., 2011). In this sense, it is well documented that in most cancer cells there is a NRF2 upregulation which acts as protective mechanism in order to promote tumor progression and chemotherapy resistance (Almeida Lima et al., 2023). Interestingly, it was demonstrated that under oxidative stress CMA is activated and promotes the degradation of KEAP1, the negative regulator of the nuclear NRF2, elevating the levels of NRF2 and inducing the transcription of several antioxidant genes, as well as LAMP2A gene expression, which further enhances CMA activity (Zhu et al., 2022).

Moreover, CMA can promote tumor progression by degrading tumor suppressors such as the nuclear receptor co-repressor (N-CoR), an essential transcriptional factor known to negatively modulate proteins involved in several oncogenic pathways (Ali et al., 2011). The degradation of misfolded N-CoR by CMA led to survival and growth of NSCLC cells, through attenuation of misfolded N-CoR-induced ER stress and possible oncogenic signaling pathways activation, what could be prevented by LAMP2A silencing in these cells (Ali *et al*., 2011). In addition, another example is the SMAD3 protein, a member of the SMAD (mothers against decapentaplegic) family that acts as an intracellular signal transducer and transcriptional factor induced by TGF-β (transforming growth factor-beta). Likewise, SMAD3 downregulation by CMA augmented proliferation and invasion of glioma cells, supporting the negative correlation between SMAD3 expression and tumor development reported in previous studies (Liu et al., 2022).

On the other hand, CMA plays a significant role as a tumor suppressor in non-tumorigenic cells. For instance, MYC degradation is dependent on the dephosphorylation at the Ser62 residue performed by the protein phosphatase 2A (PP2A) and counteracted by CIP2A. A study pointed out that CMA targets CIP2A, leading to its degradation via the ubiquitin-proteasome system, which in turn prevents MYC-driven malignant transformation of normal fibroblasts (Gomes et al., 2017 a ). Similarly, it was previously reported that hexokinase 2 (HK2), a key glycolytic enzyme upregulated in various cancer cells (Shinohara et al., 1994; Mathupala et al., 2001; Patra et al., 2013) and required for oncogenic transformation and tumor development (Patra *et al.,* 2013), undergoes degradation through CMA (Xia et al., 2015).

Furthermore, once established a tumor, CMA can slow down cancer progression by reducing the levels of tumor promoters commonly overexpressed in many tumors. For instance, CMA can degrade mutant p53, known to be involved with proliferation, resistance to apoptosis, invasiveness and migration in cancer cells (Vakifahmetoglu-Norberg et al., 2013). Another report suggested that Galectin-3 (Gal3), an anti-apoptotic and oncogenic protein, can be degraded by CMA upon c-Abl/Arg tyrosine kinases inhibition. Silencing of Gal3 and c-Abl/Arg rendered MCF7 cells more susceptible to apoptosis, resulting in reduced tumor growth (Li et al., 2010). Likewise, the epidermal growth factor receptor pathway substrate 8 (Eps8), implicated in tumor promotion and metastasis, was proposed as a CMA substrate in human cancer cells (Welsch et al., 2010).

Thus, since the first identified CMA substrate, the protein RNase A (McElligott et al., 1985), the list of formally validated or proposed CMA substrates continues to grow and includes metabolic enzymes, transcription factors, cell cycle regulators, proteins involved in the early steps of cellular translation, in cell survival or death and immune system, pro-oncogenic proteins and tumor suppressor proteins, among others.

## How the CMA gives an edge to cancer

Since several CMA targets, from tumor suppressor proteins to oncogenes, are involved in different cellular functions, the degradation of proteins by CMA in the context of cancer can offer important advantages for tumor development (Robert et al., 2019). In the initial phases of the conversion of a healthy cell to a cancerous one, specific metabolic procedures undergo modifications. These changes result in anaerobic glycolysis becoming the preferred mode for energy production rather than oxidative phosphorylation, regardless of the availability of oxygen. This preference results in increased glucose intake owing to the reduced energy efficiency of anaerobic glycolysis. Simultaneously, it triggers extensive formation of lactate and other metabolites, which have been demonstrated to boost proliferation and consequently promote tumor growth (Arias and Cuervo, 2020). This significant pro-tumorigenic occurrence was first identified by Otto Warburg in the 1920s (which is why it became known as the Warburg effect) and it has been found that CMA plays a role in promoting it (Tang et al., 2017).

In lung cancer and melanoma cells, it was found that CMA is essential for sustaining the Warburg effect by degrading p53 and preventing its inhibitory role on the transcription of glycolytic enzymes, such as glyceraldehyde-3-phosphate dehydrogenase and aldolase. Experimental validation occurred through the confirmation of an energy deficit following CMA blockade, resulting in decreased proliferation and increased cell death (Kon et al., 2011). Additionally, the acetylated form of the embryonic M2 isoform of pyruvate kinase, which is more prevalent in cancer cells, is selectively degraded by CMA promoting the accumulation of glycolytic intermediates that enhance cell proliferation and growth (Lv et al., 2011).

Unlike these two scenarios where tumor growth is reduced by blocking CMA - due to the decrease of glycolytic flux and/or the accumulation of glycolytic intermediates - CMA activation can, in certain cases, also cause a metabolic crisis (Tasset and Cuervo, 2016). Hexokinase-II (HK2), a crucial catalyst in glucose metabolism and necessary for tumorigenesis, is also a substrate of CMA, which is why upregulation of CMA also has been proposed as an effective strategy for inducing a metabolic crisis and subsequent cell death in cancer cells. However, the KFERQ motif of HK2 is hidden in the protein when there is a glucose molecule, hindering its degradation by CMA. Another impediment for the degradation of HK2 by CMA is the phosphorylation of this enzyme on Thr473, something that is frequent in breast cancer. This elucidates the potential of HK2 inhibition in reducing cancer cell proliferation (Xia et al., 2015; Yang et al., 2018).

Besides being involved in the execution of the Warburg effect in tumor cells, CMA also controls the levels of executors of the cell cycle, providing significant benefits to cancer cells in terms of proliferation rates (Andrade-Tomaz et al., 2020). One of the essential executors of cell cycle process that is degraded through CMA is p73, a transcription factor that has the ability to promote cell cycle arrest and induce apoptosis. A recent study by Nguyen et al. (2020) demonstrated that this degradation is mediated by the nerve growth factor receptor (NGFR). Also, the hypoxia inducible factor 1 subunit alpha (HIF-1α) is a transcription factor that is regulated by CMA and is involved in negatively regulating DNA replication under hypoxic conditions (Goda et al., 2003).

Another advantage that CMA can offer by degrading a substrate involved in the cell cycle, such as CHK1, is that it prevents cell cycle arrest in G2 in response to DNA damage, which leads to heightened susceptibility to genotoxic stress, as CHK1 accumulates and DNA damage increases (Park et al., 2015). CMA also regulates the Rho family GTPase (RND3), which can hinder proliferation through control of the cell cycle. RND3 decreases MYC’s transcriptional activity and expression. This reveals the vital role of CMA in the cell cycle machine, as MYC impacts various gene regulators of the cell cycle, such as cyclins, cyclin-dependent kinases (CDKs) and EF2 transcription factor (Andrade-Tomaz et al., 2020).

As well as having an impact on cell cycle and consequently on cell proliferation, CMA is also capable of affecting the migratory capacity of tumor cells and promoting their dissemination (Arias and Cuervo, 2020). A recently published study highlights YAP1 (yes-associated protein 1) and IL6ST (interleukin 6 cytokine family signal transducer) as novel targets of CMA. Both substrates are related to an increase in cell migration besides proliferation in human hepatocellular carcinoma (HCC) and hepatocyte cell lines. The knockdown of LAMP2A led to increased cell proliferation and migration in these cell lines, likely as a result of upregulated YAP1 and IL6ST, providing evidence for the tumor-suppressive effect of CMA (Desideri et al., 2023). Conversely, CMA has also been implicated in promoting tumor metastasis in lung and breast cancer cells (Kon et al., 2011; Han et al., 2017). The molecular mechanisms behind CMA-dependent metastasis remain largely unknown, but studies indicate that the degradation of the HSD17B4 protein by CMA is responsible for this invasive and migratory property (Arias and Cuervo, 2020).

Therefore, it is clear that CMA emerges as an indispensable mechanism in the development and progression of cancer cells as it offers essential edges for these cells. Studies using animal models support this finding, as mice with selective hepatic CMA inhibition displayed a higher incidence of spontaneous hepatic tumors with age (Hubert et al., 2022). But perhaps the greatest impact of CMA activity is revealed after the onset of cancer, since CMA is closely related to the development of resistance to cancer therapy.

## The role of the CMA in cancer treatment

### CMA and chemotherapy

The most prevalent approaches to treat cancer encompass surgical procedures, radiation therapy, and chemotherapy, with the specific treatment modality being determined by cancer type and its severity (Mansoori et al., 2017). Despite considerable advancements in cancer research, chemotherapy remains a promising option for cancer treatment. Nevertheless, a significant number of cancerous cells develop resistance to chemotherapeutic drugs, which significantly contributes to tumor progression and recurrence. This resistance currently stands as the main limiting factor in 90% of metastatic cancer treatment, impairing the chances of cure from this disease. Consequently, it is of extreme importance to further investigate drug resistant cancers to facilitate the development of novel therapeutic interventions (Emran et al., 2022).

Drug resistance represents a multifaceted challenge, as cancers can develop resistance via different mechanisms (Holohan et al., 2013; Vasan et al., 2019). Chemotherapeutic agents increase damage on various cellular components, promoting the accumulation of misfolded proteins and damaged organelles. Paradoxically, instead of inducing the death of cancer cells, these molecules can be degraded into substances that may sustain metabolism and support further growth and survival of tumor cells via the autophagy pathway. This process ultimately promotes resistance to therapeutic drugs. The mechanisms underlying resistance can be classified into two categories: one linked to high basal autophagy flux in certain tumor cell types, resulting in intrinsic resistance to chemotherapy, and the other associated with a gradual increase in autophagic flux in response to prolonged chemotherapy, thereby promoting acquired drug resistance (Lippert et al., 2008; Yang et al., 2022).

Extensive research has established a connection between drug resistance and upregulated autophagy (Sui et al., 2013). Beyond its role in eliminating damaged organelles, autophagy flux can serve as a cellular mechanism for overcoming environmental stress, acting as a protective mechanism that promotes tumor growth (Sui *et al*., 2013). In certain cases, the selectivity of CMA can further contribute to drug resistance since some proteins can only be transported and degraded via this pathway (Rios et al., 2021). Notably, elevated levels of LAMP2A have been associated with poor survival rates in non-small cell lung cancer (NSCLC) (Ichikawa et al., 2020). The blockade of CMA has been identified as a key driver of resistance, primarily due to its involvement in the modulation of several factors involved in the regulation of transcription, translation, and cell cycle control (Andrade-Tomaz et al., 2020). Consequently, managing the CMA pathway may hold promise as a therapeutic approach, particularly for patients who are ineligible for surgery and depend only on chemotherapy and radiotherapy.

Proof of concept for the therapeutic potential of targeting CMA with chemotherapeutic drugs has been demonstrated across various cancer types through the genetic modulation of LAMP2A. Recent studies have revealed that LAMP2A regulates malignancy by regulating apoptosis, and the suppression of LAMP2A enhances the sensitivity of cancer cells to several drugs, such as cisplatin, doxorubicin, 5-fluorouracil, bortezomib, among others (Huang et al., 2016; Karagounis et al., 2016; Ichikawa et al., 2020). On the other hand, downregulation of CMA activity can be sufficient to increase HIF-1α levels and lead to temozolomide resistance in glioblastoma (Lo Dico et al., 2018, 2021), indicating a dual role of CMA in cancer, which needs to be further investigated in order to overcome drug resistance. 

It is important to note that the term “resistance” is typically used to describe stable alterations in the cell (such as mutations) that alter the cellular response. However, emerging evidence suggests that non-genetic modifications, such as transient phenotypic adaptations, also play a role in the acquisition of resistance to chemotherapy, even though these adaptations are more generally referred to as “tolerance” mechanisms (Salgia and Kulkarni, 2018). Given this inconsistency in nomenclature, adaptations in CMA activity in response to the tumor microenvironment, which are related to the level of expression of LAMP2A rather than mutations in its gene, that could be considered involved in the development of “tolerance” to treatment are here described as triggers of “resistance” to chemotherapy, noting the predominance of the use of this term in the studies that investigated the effect of CMA in response to treatment and are summarized in Table 2.

**Table 2 -:** CMA impact on cancer treatment.

Treatment	Cancer type	Cell lines	Effect of CMA	Modulation type	Ref.
Cisplatin	Esophageal squamous cell carcinoma Non-small cell lung cancer	ESCC KYSE, A549, H460, H226, PC9, PC14, H1299	CMA activity contributes to cisplatin resistance.	shRNA, siRNA	Karagounis et al. (2016) Ichikawa et al. (2020) Cao et al. (2021)
Doxorubicin	Breast cancer Lung cancer Liver cancer	MCF-7, T47D, A549, H1299, Hep3B, Mahlavu, J7	CMA activity leads to doxorubicin resistance.	siRNA, shRNA, gene overexpression, pharmacological inhibition	Saha (2012) Karagounis et al. (2016) Huang et al. (2020)
Temozolomide	Glioblastoma	U251, U87, T98G	CMA activity contributes to sensitivity to temozolomide through HIF-1α upregulation.	siRNA	Goda et al. (2003) Lo Dico et al. (2019)
5-fluorouracil	Colorectal cancer	HCT-116, DLD-1	Increased CMA activity contributes to the activation of the NF-kB pathway and the expression of PDL-2, contributing to 5-FU resistance.	shRNA, gene overexpression	Xuan et al. (2021)
Radiotherapy	Hepatocellular carcinoma Prostate cancer Lung cancer	SMMC7721, HepG2, Hep3B, DU145, PC3, A549, H1299	Intensified autophagic flux, marked by CMA, favors irradiation-generated stress control and consequent radiotherapy resistance.	shRNA, siRNA	Koukourakis et al. (2015) Karagounis et al. (2016) Wu et al. (2017)
Photodynamic therapy	Human cervical carcinoma Rat bladder carcinoma	HeLa, AY27, mouse embryonic fibroblasts (MEFs)	CMA promotes resistance to PDT treatment by protecting cells from ROS-induced injuty.	siRNA	Dewaele et al. (2011)

*Cisplatin and CMA*

Cisplatin is a well-established chemotherapeutic agent that forms covalent bonds with DNA bases, resulting in DNA adducts. It induces diverse DNA lesions that block transcription and replication, triggering intricate intracellular signaling cascades in an effort to eliminate these lesions. In cases of compromised repair mechanisms or excessive damage, the cells undergo apoptosis (Dasari and Tchounwou, 2014). Cisplatin has exhibited notable efficacy against a broad spectrum of solid tumors, including testicular, ovarian, lung, bladder, cervical, and head and neck neoplasms (Dasari and Tchounwou, 2014). Nevertheless, occurrences of treatment-related side effects as inherent and acquired resistance persist as a significant hurdle in cisplatin-based anticancer therapy, posing a challenge throughout the treatment cycles (Rocha et al., 2018).

Damaged or misfolded proteins resulting from cisplatin activity can be recognized by LAMP2A and subsequently translocated for degradation in lysosomes (Cuervo and Wong, 2014). In fact, LAMP2A levels were found to be elevated in most cisplatin resistant cells, indicating that high CMA activity can be considered as a predictive factor for the resistance to platinum-based chemotherapy. Thus, it was observed that CMA blockade conferred cisplatin therapeutic advantages to lung cancer cells *in vitro* and *in vivo*, leading to higher cleaved caspase-3 and lower cyclin D (Karagounis et al., 2016; Ichikawa et al., 2020). CMA inhibition also overcame cisplatin resistance in esophageal squamous cell carcinoma cells (Cao et al., 2021).

*Doxorubicin and CMA*

Doxorubicin, an anthracycline widely used in treating several solid tumors, exerts its anticancer effects by intercalating with DNA or covalently binding to proteins involved in DNA replication and transcription. It leads to protein synthesis inhibition and apoptosis induction. It also interacts with mitochondrial DNA, disrupting essential mitochondrial functions (Yang et al., 2014). Recent studies have indicated that doxorubicin initiates cellular changes consistent with autophagy induction (Koleini and Kardami, 2017), and CMA has been identified as playing a pivotal role in doxorubicin resistance (Huang et al., 2020; Saha, 2012).

In HCC, doxorubicin is broadly used, however, patients often develop resistance. Notably, long non-coding RNA FAM215A has been found to interact with LAMP2A, preventing its ubiquitination in HCC cells, thereby promoting the accumulation of LAMP2A and high CMA activity, which leads to doxorubicin resistance. LAMP2A has been associated with tumor growth and recurrence in HCC, and its downregulation has been shown to reduce proliferation and viability upon doxorubicin treatment (Huang et al., 2020). Doxorubicin is also commonly employed in the treatment of early-stage, node-positive, HER2-positive, and metastatic breast cancer. Overcoming drug resistance and minimizing toxicity in this type of cancer has proven to be particularly challenging. It has been shown that breast cancer cells deficient in LAMP2A have increased sensitivity to doxorubicin, marked by higher levels of reactive oxygen species (ROS) and apoptosis compared to wild-type cells (Saha, 2012).

*Temozolomide and CMA*

Temozolomide (TMZ) is a FDA-approved oral alkylating agent for use as a first-line treatment for glioblastoma multiforme. TMZ effectively crosses the blood-brain barrier and methylates purine bases of DNA (specifically O6-guanine, N7-guanine, and N3-adenine), ultimately leading to cell death. While TMZ has extended median patient survival rates, treatment failure has been largely associated with tumor drug resistance. Notably, in contrast to previously mentioned chemotherapeutic drugs, the downregulation of CMA has been identified as a significant contributor to resistance against TMZ in gliomas (Zhang et al., 2012; Ortiz et al., 2021). In fact, it was demonstrated that CMA plays a pivotal role in the degradation of HIF-1α, a factor directly associated with glioma malignancy and resistance (Lo Dico et al., 2018, 2021). Furthermore, the silencing of HSC70 or PHLPP1 has also led to resistance characteristics in TMZ-treated cells, similar to the outcomes observed in LAMP2A-silenced cells. Interestingly, mitochondrial ROS release induces CMA activation, which is essential for the toxicity caused by TMZ (Lo Dico *et al*., 2019).

*5-fluorouracil and CMA*

Among the different types of chemotherapeutic agents, those with antimetabolic activity are popular, specially 5-fluorouracil (5-FU), which acts as a thymidylate synthetase inhibitor. 5-FU is considered an important treatment option for colorectal cancer (CRC), however, the development of a resistance is quite frequent (Vodenkova et al., 2020). In this matter, it was demonstrated that elevated CMA activity can be related to loss of sensitivity to 5-FU in *in vitro* studies with CRC cells, being that process strongly related to the activation of the NF-kB pathway and the resulting enhanced production of PLD2, an enzyme associated with tumor progression and worse prognostics (Xuan et al., 2021). In that sense, it was observed that cell lines with higher LAMP2A concentrations presented faster and increased growth, as well as elevated PLD2 expression and were more resistant to treatment with 5-FU (Xuan *et al.,* 2021).

### CMA and radiotherapy

Although chemotherapy constitutes an important facet of cancer treatment, it is not the sole one, the establishment of combinatorial treatment schemes involving chemotherapy and radiotherapy is frequent. Irradiation seeks to induce cell death, as well as suppresses tumor growth by inducing DNA damage in cancer cells through the application of high doses of radiation. Radiotherapy is one of the first-line treatments for solid cancers, such as lung, breast and esophageal cancer. However, evasion mechanisms can lead to the emergence of resistance and consequent unsuccessful treatment, as well as potential disease recurrence (Wu et al., 2023).

In this scenario, autophagic processes constitute an important tool for the onset of resistance, as they allow the disposal of the various damaged structures generated in the irradiation process. Based on an analysis of LAMP2A and LC3A levels, markers for lysosomes and autophagosomes respectively, a study showed that the presence of a more intense autophagic activity favored a radiotherapy-resistant phenotype in prostate carcinoma cells (Koukourakis et al., 2015). Similar results were observed in a study that analyzed the same markers, as well as LC3B, p62, TFEB in lung cancer cell lines (Karagounis et al., 2016).

In addition, an *in vitro* study using HCC cell lines showed that this process could favor resistance to irradiation due to the negative modulation of HMGB1. This protein would bind to LAMP2A and then be degraded by the CMA pathway, which led to a reduction in p53, an extremely important factor in inhibiting tumor growth (Wu et al., 2017).

Given presented results, the inhibition of LAMP2A has been pointed out as an alternative to sensitizing neoplastic cells to radiotherapy and increasing treatment efficiency.

### CMA and photodynamic therapy

Another promising anticancer treatment is photodynamic therapy (PDT), which has been approved by the FDA and is currently undergoing numerous clinical trials. PDT is a treatment that combines light and a photosensitizer to generate highly cytotoxic ROS, primarily in the form of singlet oxygen. These ROS react with cellular molecules, ultimately leading to organelle damage and cell death. To generate ROS, PDT employs photosensitizers (PS) that are excited by visible light at power levels that do not harm healthy tissue (Bartusik-Aebisher et al., 2021). Given that CMA functions as an effective defense mechanism against ROS-induced injury, it has been observed that PDT treatment triggers the recruitment of the CMA machinery to lysosomes in photosensitized cells. Inhibition of LAMP2A significantly increased the sensitivity of mouse embryonic fibroblasts to a wide range of PDT doses. Additionally, LAMP2 deficiency contributed to the activation of caspase-3 and cleavage of PARP, indicating a crucial mechanism of resistance to PDT via CMA activity (Dewaele et al., 2011).

## The challenge of specific CMA modulation

Despite the numerous connections between CMA and cancer biology, and the aforementioned evidence suggesting that modulation of CMA holds great potential for improving the therapeutic response of various types of cancer, the absence of chemical selective modulators for CMA presents a challenge to the therapeutic translatability of these findings, hindering the application of these positive results in clinical practice.

Until now, studies investigating the therapeutic value of CMA modulation in the context of cancer have relied solely on genetic modulation of LAMP2A to establish this proof of concept, however such approach faces significant experimental and clinical limitations (Arias and Cuervo, 2020). Even in *in vitro* assays, an important limitation of the genetic modulation of LAMP2A is that it does not allow the study of acute inhibition of CMA. This is due to the lengthy half-life of the protein, which mandates a wait time of at least five days for a significant reduction in LAMP2A levels to occur (Patel and Cuervo, 2015). Thus, despite the significant progress made within gene therapy, it faces hindrances in terms of technical, ethical, political, and financial factors (Das et al., 2015; Shahryari et al., 2019). 

Although it is evident that finding chemically specific modulators of CMA is a top priority, there are still numerous challenges to overcome to achieve this goal. One of the primary unknowns that makes the development of effective and targeted chemical compounds very challenging is the lack of information about the timeline of CMA, which transitions from a physiological and protective mechanism to a detrimental and potent pro-tumorigenic (Hubert et al., 2022). Nevertheless, the main obstacle for this development is the lack of exclusive and “druggable” compounds for CMA. This is because most of the key compounds in this pathway are multifunctional proteins that are involved in other essential cellular processes, often leading to significant levels of toxicity (Anguiano et al., 2013). For instance, the blockage of hsc70 also impacts other crucial mechanisms of protein folding and aggregation, such as e-MI, MA, and endocytosis (Patel and Cuervo, 2015).

The most exclusive component of CMA is LAMP2A and, therefore, this protein is usually targeted in studies involving CMA (Kaushik and Cuervo, 2018). However, due to the high homology (nearly 85%) of LAMP2A with the other splicing variants of the *LAMP2* gene (LAMP2B and LAMP2C) it makes a difficult target for chemical modulators that do not have the same precision as gene modulation techniques. So, such modulators may act non-specifically on the other isoforms and therefore affect other cellular functions, including MA, biogenesis, and cholesterol trafficking (Massey et al., 2006; Valdor et al., 2014).

### CMA inhibitors

Inhibiting CMA activity is a potential therapeutic approach in cancer as CMA is abnormally upregulated in many cancers and required for optimal tumor growth and metastasis (Li et al., 2018). As it is evident from Table 2, drug resistance in cancer cells can often be overcome through CMA inhibition. Unfortunately, the absence of a selective chemical inhibitor of CMA is the major barrier for translating these experimental findings into treatments for oncological patients (Du et al., 2017). Thus, no clinical trial has yet selectively targeted CMA for the treatment of any cancer, that is we still face many challenges in finding drugs that can selectively modulate CMA to maximize therapeutic effects and minimize toxicity in clinical use.

The main challenges relie in designing selective molecules that comprise only the CMA activity, without affecting other autophagic pathways. Once, inhibiting MA under certain conditions may cause tumorigenesis and metastasis, it is crucial to ensure that no such adverse effects occur (Han et al., 2017). Thus, although the initial screening studies of Finn et al. (2005) identified molecules capable of inhibiting the CMA process, including cycloheximide and anisomycin, it has already been demonstrated that the activity of these protein synthesis inhibitors is unsuitable for specifically studying the effect of CMA inhibition (Finn *et al*., 2005; Patel and Cuervo, 2015).

The latest study to search for a CMA inhibitor has identified Polyphyllin D (PPD) as a compound that inhibits the interaction between hsc70 and LAMP2A, as well as the homomultimerisation of LAMP2A, which limits tumor growth in NSCLC cells. Nevertheless, PPD exhibited an impact on MA as it obstructs the STX17-SNAP29-VAMP8 signaling pathway, which prevents a compensatory regulation of MA after the inhibition of CMA. Hence, it cannot be deemed selective for CMA (Dong et al., 2023).

Overall, most articles concerning the chemical inhibition of CMA use compounds that aim to target the proteolytic activity of lysosomes. Due to this, other forms of autophagy, in addition to CMA, are also disrupted. It is worth noting that recent clinical interventions have concentrated on using hydroxychloroquine, an intralysosomal proteolysis-inhibiting compound that alters the pH of the lysosome, as a means of intervening in the MA pathway with the aim of anti-cancer strategies. Although the degradation of substances by CMA once they are internalized in the lysosomes is not limited by pH, the continued rise in lysosomal pH results in destabilizing the luminal form of hsc70, that is vital for substrate translocation to the intralysosomal compartment. Consequently, hydroxychloroquine also leads to the inhibition of CMA. Therefore, future research should focus not only on finding the first chemical selective inhibitors of CMA but also on gaining a better understanding of how CMA blockage contributes to the beneficial effects seen in clinical trials with classical MA inhibitors, like hydroxychloroquine (Wang and Mao, 2014; Arias and Cuervo, 2020). Since the individual contribution of macroautophagy and CMA to the overall involvement of autophagy in the response to chemotherapy in clinical level remains unclear due to the absence of specific modulators for CMA.

## Conclusions

This review highlighted the great progress made in understanding the mechanisms underlying CMA over the past decades. We have discussed the primary methods available for studying it, the characteristics of the tumor microenvironment that promote CMA modulation, the proteins regulated by CMA that are pivotal for cancer development, the advantages that CMA can confer on neoplastic cells, and specially, the interplay between CMA and the development of therapy resistance in cancer. As our knowledge regarding this topic increases, it has become clear that CMA’s modulation may improve the therapeutic response to various types of cancer grows in direct proportion. However, without a doubt, the biggest obstacle in CMA modulation to be translated into clinics the scarcity of selective chemical modulators of this pathway.

In this scenario, the presented subject still has a long way to improve in order to reflect the knowledge developed in the sphere of basic research into proper clinical treatments regarding the modulation of autophagy, going beyond chloroquine. This process constitutes a possible alternative treatment scheme for patients who face resistance to traditional chemotherapy agents. Therefore, CMA is an emerging and exciting research area, that holds potential to be an alternative route to improve cancer treatment.
